# Daraxonrasib in metastatic pancreatic cancer (RASolute 302): survival breakthrough or real-world access barrier?

**DOI:** 10.1097/MS9.0000000000005214

**Published:** 2026-06-03

**Authors:** Muhammad Qaseem, Esha Khan, Abdul Haseeb Hasan

**Affiliations:** a Department of Medicine, Khyber Medical College, Peshawar, Pakistan; b Department of Medicine, Nishtar Medical University, Multan, Pakistan; c Department of Research, Oli Health Magazine Organization, Kigali, Rwanda; d Department of Internal Medicine, Sentara Northern Virginia Medical Center, Woodbridge, VA, USA; e Department of Medicine, King Edward Medical University, Lahore, Pakistan

*To the Editor,*

Pancreatic ductal adenocarcinoma (PDAC) is a malignant disease of ductal epithelial origin, characterized by rapid progression and high metastatic rates. Oncogenic RAS mutations (>90% of cases) that result in constitutive guanosine triphosphate (GTP)-bound RAS activation cause persistent MAPK/ERK and PI3K/AKT signaling, which is the primary cause of pathogenesis in these tumors. It presents with a clinical picture of epigastric/back pain, jaundice, weight loss, and fatigue. Between 1990 and 2021, global incidence rose from 207 905 to 508 533 cases, with age-standardized incidence rate increasing from 5.47 to 5.96 per 100 000; disability adjusted life years rose from 5.21 million to 11.32 million, and deaths increased from 211 613 to 505 752[1]. An essential fact that has been under-acknowledged is the virtually universal RAS addiction in PDAC, as well as in RAS-mutant non-small cell lung cancer (NSCLC) and colorectal cancer (CRC). Existing therapies are based on chemotherapy, such as FOLFIRINOX. A pan-RAS (ON) inhibitor, daraxonrasib, has emerged as a promising cornerstone.

Daraxonrasib (RMC-6236) is a preclinical oral, non-covalent, multi-selective RAS(ON) tri-complex, an inhibitor of the active GTP-bound form of mutant and wild-type RAS isoforms (G12X, G13X, and Q61X). Its action pathway is to develop a high-affinity tri-complex with cyclophilin A and RAS(ON), which directly inhibits effector binding (e.g., RAF) and potently inhibits downstream MAPK and PI3K signaling, regardless of mutation subtype. Daraxonrasib showed a 60% decrease in the risk of death with a median overall survival of 13.2 months versus 6.7 months with standard chemotherapy in the pivotal Phase 3 RASolute 302 trial in previously treated metastatic PDAC (hazard ratio 0.40, *P* < 0.0001), along with significant improvement in progression-free survival and a manageable safety profile. It provides easy oral dosing (300 mg) once a day and wide genotype coverage[2]. First-line and later-line RAS-mutant metastatic PDAC therapies have the potential to be extended to RAS-mutant NSCLC and CRC.

Robust clinical evidence supports the efficacy of daraxonrasib. In a phase 1/1b study on monotherapy (NCT05379985), PDAC patients with prior treatment for their RAS mutant cancer, receiving active drug doses between 160 and 300 mg, showed a response rate of 29% [95% confidence interval (CI): 16–45] for individuals with KRAS G12X mutations (*n* = 42) and 25% (95% CI: 14–38) for those with RAS mutations (*n* = 57). The median progression free survival was 8.5 months (95% CI: 5.3–11.7) and 7.6 months (95% CI: 5.9–11.1) for both groups, respectively. Molecular responses reflected a >50% reduction in the RAS mutant circulating tumor DNA variant allele fraction in 95% (53/56) of patients with KRAS G12X and 93% (63/68) of those with RAS mutations[3]. Updated studies also support the continuing engagement of targets and antitumor effects in different RAS mutations among patients with PDAC who have received prior therapy[4].

Despite the proven efficacy of daraxonrasib, there are problems that prevent its widespread adoption due to challenges associated with practical implementations. Although it has been shown to increase patient survival by almost twice as much in those who have undergone the treatment process for metastatic PDAC, the high costs and unpredictable reimbursement programs become obstacles to implementation, especially in low- and middle-income countries where the rate of pancreatic cancer is increasing. Specialized centers for molecular diagnosis of RAS genotyping, as well as proper preparation for handling side effects and integration within the therapy process, are required for the treatment of this condition. Challenges such as delays in the approval process, lack of testing on different demographics, and geographic differences further prevent widespread access. All these barriers pose risks of developing inequalities between groups in terms of outcomes, since underprivileged populations have a higher mortality rate from pancreatic cancer yet face issues related to high costs and insurance[5].

In summary, the persistent global challenge posed by RAS-induced solid tumors, particularly the highly aggressive nature of pancreatic cancer, where almost all patients exhibit RAS mutations, emphasizes the significance of applying daraxonrasib optimally. Although its potential effectiveness as a revolutionary therapeutic intervention is well-documented, the current limitations in practical settings restrict its impact on many individuals. Maximizing the gains in survival rates associated with RAS-mutant tumors such as PDAC, NSCLC, and CRC requires improving detection methods, bringing healthcare services closer to patients, and reducing costs.

## Data Availability

Data sharing is not applicable to this article, as no datasets were generated or analyzed during the current study.
